# Multi-faceted role of LRP1 in the immune system

**DOI:** 10.3389/fimmu.2023.1166189

**Published:** 2023-03-20

**Authors:** Olga Sizova, Lisa St. John, Qing Ma, Jeffrey J. Molldrem

**Affiliations:** ^1^ Department of Hematopoietic Biology and Malignancy, Division of Cancer Medicine, The University of Texas MD Anderson Cancer Center, Houston, TX, United States; ^2^ ECLIPSE, Therapeutic Discovery Division, The University of Texas MD Anderson Cancer Center, Houston, TX, United States

**Keywords:** LRP1, CD91, LDL receptor family, innate immunity, adaptive immunity, GvHD

## Abstract

Graft versus host disease (GVHD) represents the major complication after allogeneic hematopoietic stem cell transplantation (Allo-SCT). GVHD-prone patients rely on GVHD prophylaxis (e.g. methotrexate) and generalized anti-GVHD medical regimen (glucocorticoids). New anti-GVHD therapy strategies are being constantly explored, however there is an urgent need to improve current treatment, since GVHD-related mortality reaches 22% within 5 years in patients with chronic GVHD. This review is an attempt to describe a very well-known receptor in lipoprotein studies – the low-density lipoprotein receptor related protein 1 (LRP1) - in a new light, as a potential therapeutic target for GVHD prevention and treatment. Our preliminary studies demonstrated that LRP1 deletion in donor murine T cells results in significantly lower GVHD-related mortality in recipient mice with MHC (major histocompatibility complex) -mismatched HSCT. Given the importance of T cells in the development of GVHD, there is a significant gap in scientific literature regarding LRP1’s role in T cell biology. Furthermore, there is limited research interest and publications on this classical receptor molecule in other immune cell types. Herein, we endeavor to summarize existing knowledge about LRP1’s role in various immune cells to demonstrate the possibility of this receptor to serve as a novel target for anti-GVHD treatment.

## Introduction

Allogeneic hematopoietic stem cell transplantation (AHSCT) is a treatment for hematological malignancies by transferring stem cells from a healthy person (the donor) to the patient (the recipient) after high dose chemotherapy or radiation. Graft versus host disease (GVHD) is one of the major causes of morbidity and mortality in patients undergoing AHSCT (1). During GVHD, T cells of donor origin identify antigens on recipient cells as foreign, thus targeting recipient tissues for destruction. Major and minor human leukocyte antigens (HLA) which present self and foreign antigens, play a significant role in GVHD pathogenesis. Usually, a patient receiving an allogeneic stem cell transplant will undergo some type of GVHD prevention (2, 3). The main principle for GVHD prevention and treatment/management is a suppression of T cell activity to avoid recipient tissue damage. Immunosuppression with corticosteroids is the first-line therapy in both acute and chronic GVHD (aGVHD and cGVHD, respectively), resulting in condition improvement of less than 50% in patients with aGVHD and only 40–50% in patients with cGVHD depending on initial disease stage (4).

Studies of the pathophysiological mechanisms of GVHD, as well as deeper understanding of T cell activation, have laid a foundation for novel therapeutic approaches in GVHD prophylaxis and management (2). Due to the relatively poor efficacy of current GVHD treatments, it is essential to explore novel molecules that have the potential to be exploited to generate new, innovative anti-GVHD therapies.

The low-density lipoprotein receptor-related protein (LRP1) is a member of the LDL receptor family. LRP1 plays diverse roles in multiple biological processes including lipoprotein metabolism, endocytosis, cell growth, cell migration, inflammation, and apoptosis. It is also involved in the regulation of platelet derived growth factor receptor, vascular tone, and blood brain barrier (BBB) permeability. Additionally, LRP1 is implicated in conditions such as neurodegenerative diseases, atherosclerosis, and cancer (5–7).

Being ubiquitously expressed, LRP1 can also be found on the surface of both innate and adaptive immune cells. The ability of LRP1 to bind multiple functionally-unrelated ligands, enables the protein to direct distinct functions in different types of immune cells (8). While expression of LRP1 is abundant on myeloid cells, it is poorly expressed on the surface of lymphocytes. In innate immune cells, such as macrophages and monocytes, LRP1 is mainly known as a modulator of inflammation, where it serves a dual role, both promoting and inhibiting inflammatory responses (9–11). In T- and B- lymphocytes, the role of LRP1 is largely unknown. Due to its low surface expression, it has been much more challenging to study its role in these cells. Recently, we report that LRP1 plays an important role in human T cell proliferation, and leukemia associated antigen Proteinase 3 can modulate immunity *via* direct interaction with LRP1 on T cells (12). Furthermore, we found a novel role for LRP1 in mouse model of GVHD, and T cells deficient in LRP1 results in tolerance with incomplete donor chimerism and prevention of GVHD (13).

This review is the first attempt to describe the connection LRP1 has to the immune system, with emphasis on each immune cell type.

## LRP1 overview

The low-density lipoprotein receptor related protein-1 (LRP1) was first described through homology cloning with the LDL (low-density lipoprotein) receptor by Herz in 1988 (14). Based on the identification of specific sequence motifs within the protein, Herz et al. conjectured a role for LRP1 in the regulation of growth factor activity by way of proteolytic processing. Even during the initial characterization of LRP1, it was apparent this protein warranted further study.

LRP1 is now readily identified as a member of the LDL receptor family. This family of proteins contains structurally homologous receptors that are composed of uniformed structures. This receptor family currently consists of seven family members that are closely related, which includes the LDL receptor, very-low-density lipoprotein (VLDL) receptor, apoE receptor 2 or LRP8, multiple epidermal growth factor-like domains 7 (MEGF7), megalin or LRP2, LRP1, and LRP1b (15). Nevertheless, these proteins demonstrate some differences in their affinity to ligands, endocytosis rate, cellular localization etc., that may have an impact on their physiological roles.

LRP1 gene encodes a large 4525 amino acid protein, composed of a 515 kDa N-terminal extracellular subunit and an 85 kDa C-terminal transmembrane subunit, which are noncovalently linked with each other (16). LRP1 is a 600-kDa type I transmembrane protein processed by furin into a larger 515-kDa α- and a smaller 85-kDa β -subunit in the trans-Golgi network. To prevent premature binding of ligands to the newly synthesized LRP1, an endoplasmic reticulum chaperone - receptor-associated protein (RAP) binds to LRP1 on multiple sites enabling it to be successfully delivered to the plasma membrane (17). The resulted molecule consists of the extracellular domain also called the heavy α-chain containing four clusters (I–IV) of ligand-binding type cysteine-rich repeats (CRs) and the light β-chain (85 kDa) (14, 18). Interestingly, the β-chain provides binding sites for signaling adapter proteins, as well as various tyrosine residues, whose phosphorylation is imperative for LRP1-mediated signal transduction (17).

Due to its complex structure, LRP1 is known to bind more than thirty different ligands. LRP1 ligands include proteases, protease inhibitor complexes, extracellular matrix proteins, growth factors, toxins and viral proteins (5, 8, 19).. The ability of LRP1 to bind multiple ligands enables the protein to play a role in a number of biological processes such as lipid and glucose metabolism, protein clearance and degradation, signal transduction, maintenance of energy homeostasis, and regulation of extracellular matrix proteins. Additionally, LRP1 is involved in modulation of the blood brain barrier and neurotransmission (20–25). Furthermore, LRP1 can regulate numerous signaling pathways, including mitogen-activated protein kinase (MAPK), insulin receptor (IR), serine/threonine protein kinase (AKT), extracellular signal-regulated kinase (ERK), and c-jun N-terminal kinase (JNK) pathways. Studies of LRP1 gene deletion in various tissues and cell types acknowledge an important contribution of LRP1 in the vascular and central nervous systems, as well as in macrophages, endothelial cells and adipocytes (17, 26). The importance of LRP1 is revealed by the lethality of *lrp1* gene deletion as it terminates mouse embryo development around the implantation stage (27).

## LRP1 in cancer

Undoubtedly, the immune system and the development and/or progression of cancer are linked. Initial studies on LRP1’s possible role in cancer were mainly on tumor cell lines, and suggested that reduction of *Lrp1* expression or even a complete loss of the gene is associated with cancer development (28). However, further studies determined that LRP1 expression is substantially increased by hypoxic conditions which are common in malignancies *in vivo*. Thus, the expression of LRP1 on cancer cells cultured in ambient oxygen-rich conditions may not be an accurate reflection of its level of expression in cancer (29).

Despite non-hypoxic growth conditions, certain cancer cell lines including brain, liver, and lung do still exhibit high levels of LRP1 expression, suggesting upregulation in tumors (30). Importantly, though, numerous studies propose a dual role of LRP1 in cancer. LRP1 is reported to be upregulated in endometrial carcinomas, malignant glioma, triple negative breast cancer (31–33) and is linked with tumor metastasis and poor prognosis. In urothelial cancer and renal cancer LRP1 also serves as an unfavorable prognostic marker (34). Interestingly, tumor cell lines such as breast cancer, ovarian cancer, and melanoma do express LRP1, though at different levels, confirming that the relationship between expression of LRP1 and cancer development is complicated (35).

In contrast, inhibition of LRP1 expression in thyroid cancer intensifies the invasion ability of cancer cells to metastasize to lymph nodes and lungs (36). In this case, low cell surface expression of LRP1 is linked to high expression of urokinase-type plasminogen activator (uPA) which is known to induce tumor cell invasion (36). Supporting that, an increased expression of LRP1 results in a low metastatic potential of hepatocellular carcinoma (HCC) (37). In addition, low expression of LRP1 was reported in human endometrial carcinoma, prostate cancers, lung cancer, and Wilm’s tumors (38–41). Interestingly, there is a better overall survival of advanced melanoma patients with high LRP1 expression in monocytes (42). According to Human Protein Atlas, LRP1 in general has low cancer specificity to conclude that more studies are necessary to properly evaluate LRP1’s role in cancer (34). A recent publication demonstrated that LRP1 mediates Notch pathway activation, and knocking out LRP1 attenuates Notch-signaling-dependent invasion, migration and tumorigenesis of leukemia and breast cancer cells (43).

## Expression in mice vs. humans

Humans and mice share approximately 85 percent sequence identity in protein-coding regions. And, importantly, many genes linked to diseases in humans have counterparts in the mouse, making mice a useful research tool (44). First studies in mice reported LRP1 expression in hepatocytes, however it is known most murine cells express LRP1, including immune cells (14, 45).

Murine and human LRP1 gene have more than 98% gene sequence homology when analyzed *via* BLAST, underscoring the essential role of mouse models for studying LRP1 biology (46). LRP1 knockout mice are embryonic lethal, as the blastocysts fail to implant (27). However, studies with conditional tissue specific *lrp1* gene deletion in macrophages, dendritic cells (DCs) and T cells reveal interesting findings about the function of this protein, that are going to be discussed further.

Being ubiquitously expressed, especially with higher levels in the liver, brain and lung, LRP1 is also found on the surface of the immune cells in both mice and humans (17).

## LRP1 in the innate immune system

Innate immunity is the first line of defense against pathogens. Innate immune cells such as neutrophils, NK cells, monocytes and macrophages initiate the first quick powerful, although not specific, immune response. The innate immune cells mediate nonspecific protection through the production of pre-synthesized effector molecules or acting as direct killers of the invading cells.

A subset of innate immune cells serves as antigen presenting cells (APCs). The expression of LRP1 on APCs has been shown to enhance T- and B- cell responses by facilitating antigen uptake (47, 48). LRP1 has been shown to modulate innate immune responses by binding pseudomonas exotoxin A and rhinovirus particles (49, 50). LRP1’s role in infection processes is immense. It can assist in battling the infection by scavenging bacterial membrane lipoproteins, resulting in pathogen clearance. Additionally, it can modulate the inevitable infection-induced inflammation by inhibiting an exaggerated inflammatory response to prevent further injury (11, 51). During tissue injury, LRP1-mediated signaling can determine the fate of the cell by promoting survival and blocking the destruction of the damaged yet salvageable cells within the injured tissue (52).

## In monocytes

LRP1 is expressed on the surface of human peripheral blood monocytes, as well as monocytic cancer cell lines, such as THP1 (35, 53, 54).

### Expression in different monocyte subsets

Human monocytes are divided in three major populations that can be identified by the level of expression of CD14 and CD16 molecules. Classical monocytes are described to be CD14^+^CD16^−^, whereas intermediate are CD14^+^CD16^+^ and non-classical are CD14^dim^CD16^+^ (55). Classical monocyte population represents the majority of phagocytic cells (80–95% of circulating monocytes) and are known to play a role in scavenging. Non-classical monocytes comprise about 2–11% of circulating monocytes with their main function to survey the endothelium in search of injury. Furthermore, non-classical monocytes also play a role in promoting inflammation, antigen presentation and T cell stimulation. Intermediate monocytes represents the smallest fraction with various functions, such as production of reactive oxygen species (ROS), antigen presentation, participation in the proliferation and stimulation of T cells, inflammatory responses, and angiogenesis (56, 57). According to the Human Protein Atlas, the highest *Lrp1* gene expression is found in classical monocytes (34). Flow cytometry analysis confirms decreased expression of LRP1 in non-classical monocytes although donor heterogeneity has been noted (58). Dissimilar LRP1 expression on various monocyte populations have been linked to the development of atherosclerosis and cardio-vascular diseases (6, 59–61). LRP1 has been referred as monocyte differentiation antigen, which is expressed in different monocytic subpopulations (54, 58). It was shown that LRP1 is expressed intracellularly in these three above-mentioned subpopulation, and that 20% of total LRP1 protein is detected on the cell surface (58).

### Implication in various diseases

A number of studies have demonstrated LRP1 overexpression in monocytes from patients with long-term, non-progressing HIV infection and from subjects who were still HIV-1-seronegative regardless of exposure to the virus, implying the fact that LRP1 may be involved in protection against HIV-1 infection (62–64). An increased LRP1 expression in monocytes of hemophilic patients has also been reported (63, 65).

An increased expression of LRP1 is observed in monocytes of vitiligo patients, when compared with healthy controls. Interestingly, this phenomena is still detected after repigmentation (66). A high expression of LRP1, among other factors such as environmental conditions, may determine an individual’s susceptibility to vitiligo. A high LRP1 expression in active vitiligo patients suggests that LRP1 may play a significant role in vitiligo progression and thus LRP1 represents a potential target for novel therapeutic approaches to modulate this autoimmune condition (66).

Atherosclerosis is another disease in which peripheral blood monocytes are involved. Circulating monocytes with pro-inflammatory profiles are known to be implicated in the early stages of atherosclerosis development (67). It has been shown that LRP1 is also engaged in atherosclerosis development, specifically in atherogenic plaque formation (6). It has been proposed that decreased LRP1 expression in pro-inflammatory monocytes is linked with development of atherosclerosis (67).

## In macrophages

LRP1 is abundantly expressed in macrophages and is mainly associated with suppression of inflammation. Suppressive effects are demonstrated by the ability of the LRP1-expressing cells to engulf apoptotic cells (efferocytosis) and inhibit the production of inflammatory proteins.

### Role in efferocytosis

The phagocytosis of apoptotic cells (ACs) - efferocytosis, is critical for self-tolerance and host defense. It is one of the many functions of phagocytic macrophages, where it is driven by LRP1. It was demonstrated that LRP1 together with its coreceptor calreticulin, interact with complement factor C1q to engulf apoptotic cells (68). To support this, studies with LRP1 knockout phagocytes show that LRP1 ^−/−^ macrophages demonstrate a decreased ability to engulf ACs, when compared with wild-type cells (69). Macrophage’s LRP1, when associated with calreticulin is known to promote efferocytosis. This process is balanced by “don’t eat me signals” such as CD47. CD47 is known to be increased in a number of cancers, making tumor cells less susceptible to phagocytosis (70). Additionally, it was demonstrated that macrophage LRP1 is required for anti-CD47 blockade that results in boosting the efferocytosis, decrease of atherogenesis, and lowering necrotic core formation (71).

Mouse model of atherosclerosis is a perfect representation for LRP1-driven efferocytosis of macrophages. Under normal conditions, macrophages enter the developing plaques at the early stage of the disease. To remove the excess of the cholesterol by the liver, the cholesterol is needed to be picked up by the macrophages first. In an unfavorable condition, such as when LRP1 levels are low on the macrophages, cholesterol-overloaded macrophages can become stuck and start breaking down in the arteries. Macrophage LRP1 exports excess cholesterol out of the cell, thus lowering the risk of atherosclerosis (72). Interestingly, it has been shown that deficiency of macrophage LRP1 leads to a decreased lipoprotein internalization and thus accelerates atherosclerosis progression (73).

### Other ways of suppressing inflammation

In addition to clearance of the apoptotic cells during atherosclerosis, high expression of LRP1 on macrophage can also inhibit inflammation. This is dependent upon the dissociation of the extracellular and intracellular components of the protein. The extracellular domain of LRP1 can undergo shedding *via* the process of regulated intramembrane proteolysis (RIP), where intracellular LRP1 beta chain is released into the cytoplasm and, under certain conditions, translocates into the nucleus to regulate gene transcription. In the case of atherosclerosis, lipopolysaccharide and other inflammatory mediators specifically promote LRP1 shedding. The cytoplasmic fragment can then relocate to the nucleus, where it binds to and promotes the export of interferon regulatory factor-3 from the nucleus leading to suppression of expression of pro-inflammatory target genes, thus inhibiting inflammation (74). In addition, LRP1 is involved in inhibition of Toll-like receptor (TLR)-induced inflammation in macrophages (75). LRP1-deficient macrophages demonstrate increased expression of pro-inflammatory mediators, including TNFα and IL6 (9). Further, LRP1-deficient macrophages exposed to apoptotic stimuli have decreased AKT phosphorylation and increased expression of pro-inflammatory cytokines such as IL1β and IL6 (69). As far as LRP1 role in macrophage migration capacity, there is evidence, although limited, that LRP1 located at the plasma membrane of macrophages can participate in actin polymerization, formation of cellular protrusions, co-localizing with β1-integrins (76).

## In NK cells

LRP1 expression is not detected on murine nor human NK cells (58, 77).

## In neutrophils

LRP1 is expressed on human neutrophils, however relevant studies are very limited. LRP1 expression has been reported to increase on human neutrophils upon treatment with TNFα (78, 79). Additionally, LRP1 on human neutrophils is involved in degranulation and migration in response to recombinant tissue plasminogen activator (r-tPA) (78).

## Other innate immune subpopulations

Eosinophils, basophils and mast cells represent the rarest innate immune cell types. Based on early studies, LRP1 is not expressed in basophils (80). This observation is supported by more recent data, including from Human Protein Atlas, in which LRP1 is not detected in any of these innate immune cells (81, 82).

## LRP1 in DCs

DCs are a heterogeneous population of bone marrow-derived cells that that are involved in immunosurveillance, antigen-presentation and immune-tolerance. Microenvironment or cell culture conditions may influence on differentiation of DC subtypes. Universally, DCs comprise of the conventional dendritic cells (cDC) and the plasmacytoid dendritic cells (pDC). Dendritic cells play an important role in initiating T cell responses. LRP1 expressed on DCs is associated with activation of DCs and cross-priming of T cells (45, 83). Notably, LRP1 expression varies on different DC subsets which could affect their ability to present antigen.

### LRP1 on different DC subsets

As previously noted, different subtypes of human DCs express LRP1 at varying levels. For example, CD11c^+^ DCs with low levels of MHC Class II expression, exhibit LRP1 at higher levels compared to CD11c^+^ DCs with high MHC Class II (84). The expression of LRP1 is partially downregulated during differentiation of monocytes into monocyte-derived DCs (85). On the contrary, plasmacytoid DCs express low levels of LRP1, but in a donor-dependent manner (86, 87). LRP1 is also expressed on murine pDCs (88). Interestingly, LRP1 expression is age-dependent: it is significantly decreased in all DC subpopulations in spleens of old mice, whereas it was increased in the liver, probably due to inflammation (88). It may be explained by the fact that pDCs and CD4^−^CD8α^+^ DCs acquire lipids in various tissues during aging, which is associated with increased LRP1 expression, particularly in livers and mesenteric lymph nodes (88).

Studies with *lrp1* gene deletion in dendritic cells reveal the important role of LRP1 in these immune cell populations. It has been shown that mice deficient in LRP1 expression specifically in DCs are more susceptible to chemically induced tumors compared to wild type mice (89). With tumor progression, LRP1 deficient DCs failed to present tumor antigens to T cells to stimulate anti-cancer immune response (89).

It was also demonstrated that LRP1 acts as a negative regulator of DC-mediated adaptive immune responses in house dust mite (HDM)-induced eosinophilic airway inflammation. Furthermore, it is proposed that LRP1can be a modulator of type 2 asthma, since circulating myeloid DCs of asthmatic patients demonstrate a reduced LRP1 expression (90).

### Role in efferocytosis

Clearance of apoptotic cells through efferocytosis is imperative to impede the possibility of necrosis and further inflammation caused by the release of intracellular contents (91). Dendritic cells are also capable of efferocytosis to aid the immune system. It has been shown that LRP1 and AXL receptor tyrosine kinase cooperate to facilitate DC-mediated efferocytosis. Specifically, LRP1 is primarily involved in engulfment of AXL-bound apoptotic cells (92).

## LRP1 in adaptive immune system

In addition to the innate mechanisms of host defense there is the adaptive immune system that has specificity for its target antigens. Responses of the adaptive immune system require the interaction of antigen-specific receptors expressed on the surfaces of T- and B- lymphocytes and their cognate antigens. After recognizing and binding specific antigens, these cells can then directly or indirectly initiate the killing and/or removal of the cell expressing the antigen of interest (93).

## B cells

It is known that expression of LRP1 on B cells is very low, which makes exploration of its role in B cell biology difficult (94). It is likely that LRP1 is involved in the antigen presentation process in B cells. It has been shown that antigens chaperoned by the heat shock protein gp96 enter B cells as well as DCs through a specific, LRP1- and LOX-1-mediated mechanism, and are presented by MHC II molecules (48).

## T cells

### Adhesion and motility

LRP1 expression on steady-state T cells is very low, but this level increases upon stimulation (94–96). The low level of expression on unstimulated T cells is due to constitutive LRP1 shedding. Shedding of LRP1 plays a key role in the inhibition of T cell adhesion, which is crucial for effective function of T cells as well as maintenance of T cell surveillance. Shedding of LRP1, which is dependent on metalloproteinase ADAM10, results in the downregulation of adhesion capacity. As T cells encounter β1 and β2integrin ligands such as ICAM-1 and fibronectin and/or chemokines, specifically CXCL12, which binds to CXCR4, surface expression of thrombospondin-1 (TSP1) is induced. TSP1 has been shown to associate with LRP1 on T cells which then leads to the inhibition of LRP1 shedding. This in turn allows for induction and increased adhesion (in the case of integrin engagement) and enhanced motility (chemokine binding) (95, 96). Moreover when LRP1 is complexed with calreticulin (CRT) and TSP1, T cell adhesion is augmented by a subsequent crosslinking cascade *via* TSP1 and CD47 (97). Shedding of LRP1 from the surface of circulating T cell suppresses T cell adhesion to integrins. Thus, depletion of LRP1 from the T cell surface may promote T cell surveillance (95).

### Activation of T cells

There are no reports in the scientific literature about LRP1’s role in T cell activation. However, it has been shown that in gliomas midkine (MDK) can bind to LRP1 on tumor-infiltrating CD8^+^ T cells leading to increased CCL4 expression on T cells. This may potentially lead to further immune cell infiltration and T cell stimulation (98).

### T cell proliferation

LRP1 has been proposed to play a role in T cell proliferation. Co-culture of activated, LRP1^+^ human peripheral blood mononuclear cells (PBMCs) with proteinase-3 (P3)-expressing polymorphonuclear neutrophils (PMNs) resulted in inhibition of T cell replication. Interestingly, physical cell to cell contact is imperative for this inhibition, since using a trans-well system prevented this effect. The anti-LRP1 blocking antibody significantly reversed the inhibition and restored T cell proliferation (12).

### Indirect effect of LRP1 on T cells

LRP1 expression on other cell types may have an impact on T cell biology. LRP1 is a known receptor for a number of proteins involved in T cell activation. For example, various heat shock proteins (HSPs) bound to LRP1 can be internalized to be trafficked to MHCI for representation by APCs. These HSPs-loaded MHCI complexed can be important in further stimulation of CD8 T cells (29). As far as CD4 T cells, LRP1-induced APC maturation results in priming of CD4 T cells. Specifically, binding of HSPs to LRP1 on APCs initiates signaling cascade to activate nuclear factor kappa B, that is responsible for cytokine production. Various cytokines in the microenvironment dictate priming of specific T helper cell subsets (99).

Free unattached LRP1 has been shown to affect T cell function as well. It has been shown that the i.p. administration of LRP1-derived peptide into abortion-prone pregnant mice significantly decreased the abortion rate by increasing immunomodulating Tregs. Increased LRP1-derived peptide-Treg expansion coupled with upregulated IL2 and IL10 in mouse serum, as well as decreased expression of co-stimulatory molecules of APCs resulted in successful pregnancies in mice (100).

### Anti-leukemia immunity and allogeneic cell transplantation complications

We have been investigating the role of leukemia specific antigen Proteinase 3 (P3) in modulating anti-tumor immune response and identified LRP1 on T cells as the direct mediator of T cell proliferation (12). Membrane P3 (mP3) on acute myeloid leukemia (AML) blasts inhibits T cell proliferation through LRP1 and mP3 interaction, resulting in the evasion of anti-leukemia T cell immune response. Interestingly, LRP1 was identified as one of the genes in exome-wide association study affecting survival outcomes after unrelated-donor stem cell transplant (101). LRP1 is among the four donor genes significantly associated with overall survival (101), and 27 variants within LRP1 contributed to this association.

We generated a conditional T cell specific deletion of LRP1 (LRP1^fx/fx^LckCre^+^) mice to investigate the capacity of LRP1 in regulating T cell proliferation *in vivo* (13). LRP1-deficient T cell development is overall normal in the thymus, spleen, lymph node and blood, including no significant differences in naïve, memory, or effector subsets compared to control T cells from WT mice (LRP1^fx/fx^). Using the MHC-mismatched mouse model of GVHD, we demonstrate that LRP1 regulates alloimmune responses in GVHD. Recipient mice with LRP1-deificient T cell donor have significantly lower GVHD-related mortality and morbidity compared to WT control. The numbers of donor-derived cells and CD8 T cell subset were reduced in the spleen of recipient mice; while the percentage of naïve T cells (CD62L^+^CD44^+^) including both CD4 and CD8 subsets, and Tregs were increased. In addition, proinflammatory cytokines such as TNFα, IL17, IFNγ and IL2 were decreased in the serum of these recipient mice (13). The data support a novel role for LRP1 as a potential target for regulating T cell function and preventing GVHD.

## Discussion

**Figure 1:**
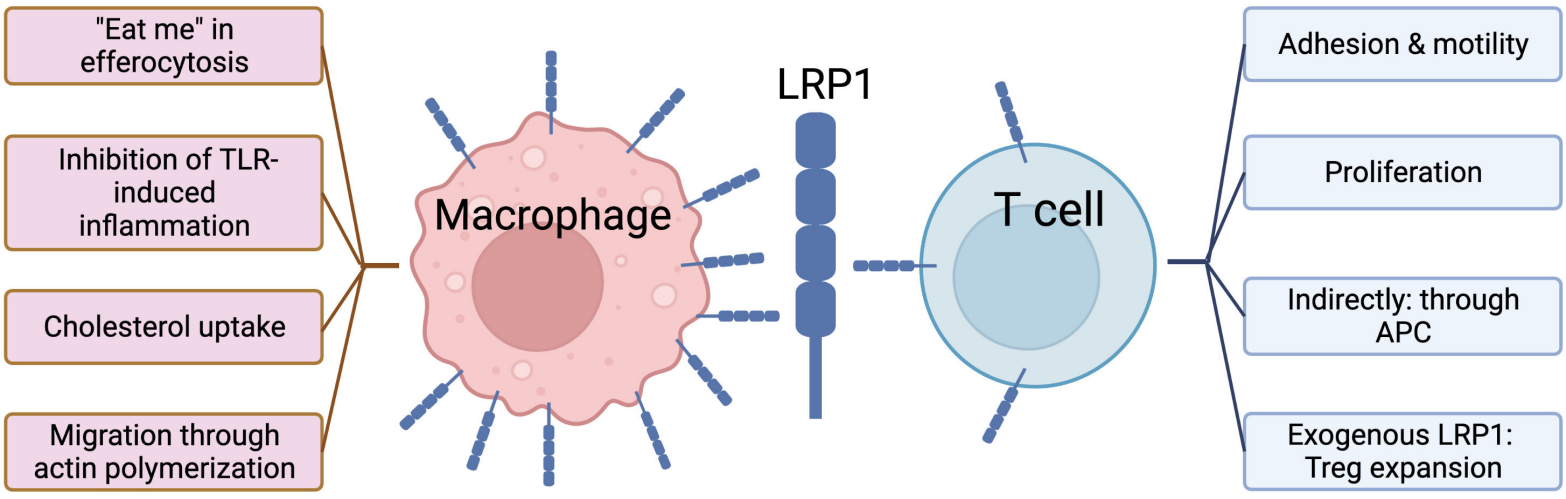
Role of LRP1 in macrophages vs. T cells.

In this review, we provide a detailed discussion of the diverse role of LRP1 in both innate and adaptive immune cell types, and how this molecule may serve as a potential target in hematological diseases. Among all immune cell subsets LRP1 has been studied most extensively in macrophages, where it contributes to its pivotal role – removal of apoptotic cells, or efferocytosis, as well as other processes (68, 69) (**Figure 1**, left panel). On the other hand, T cells – important mediators of adaptive cellular immunity – have low LRP1 expression on their surface at steady state (94–96). Recently the biology of LRP1 on T cells has drawn the attention of researchers. It is hypothesized that LRP1 may play an essential role in various T cell functions, such as proliferation, motility/adhesion balance, activation etc., that is summarized in **Figure 1**, right panel.

GVHD development and progression involves various immune subpopulations. Since T cells are the main mediators of GVHD, adaptive immunity is a key to this process. However, there is clear evidence that the innate immune system is an irrevocable counterpart to adaptive immunity during the course of GVHD, highlighted by the fact that the first step of GVHD initiation is stimulation of host APCs, a part of the innate immune system. Additionally, there is evidence that recognition of damage- and/or pathogen-associated molecular patterns during GVHD can lead to activation of neutrophils, macrophages and monocytes that results in GVHD aggravation (102). The link between innate immune receptor polymorphisms and GVHD severity was observed in patients receiving allogeneic HCT (103). As data mounts, it has become obvious that immune cells from both the adaptive and innate immune system branches are implicated in GVHD. Because LRP1 has been shown to play a significant role in orchestrating both activation and inhibition signals in various cells of both the innate and adaptive immune systems, we believe that this molecule can serve as an important target for exploration of novel anti-GVHD treatment strategies.

**Figure 2:**
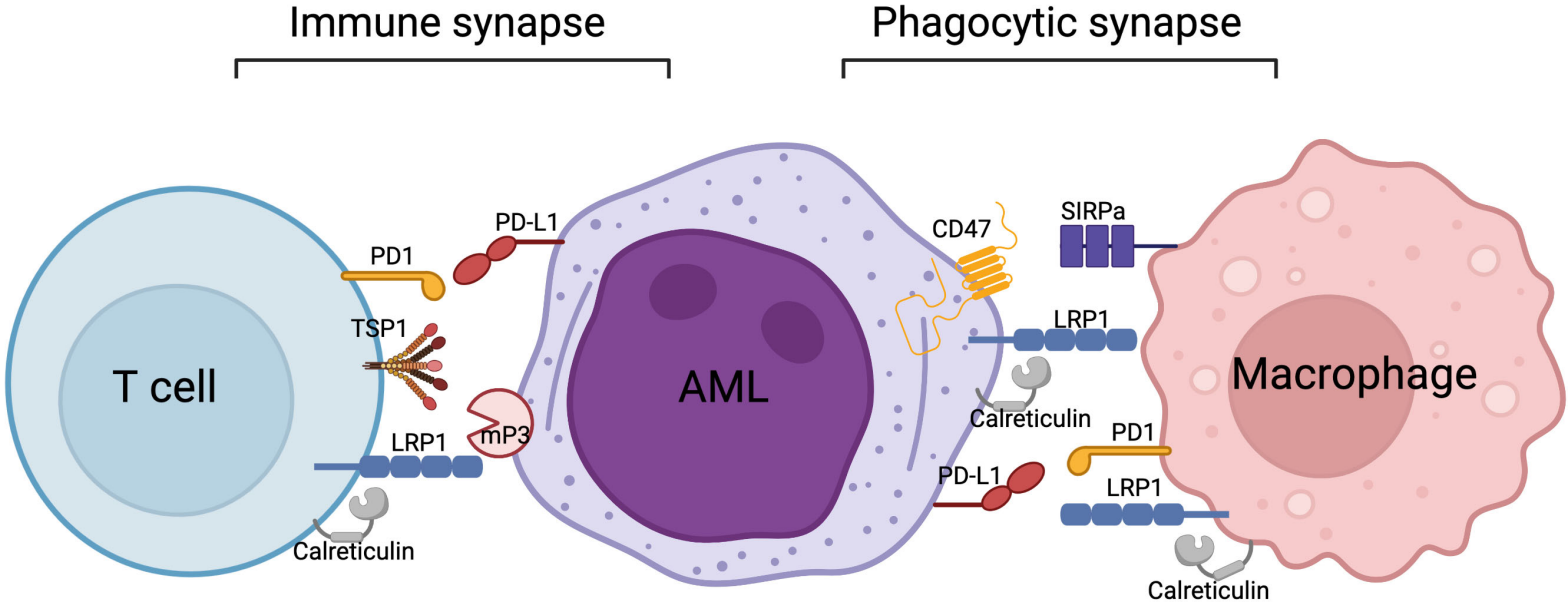
Proposed molecular interrelationships between a T cell, AML cell and macrophage.

We propose the potential molecular interrelationship between a T cell, acute myeloid leukemia (AML) cell and macrophage in **Figure 2**. LRP1 in association with its ligand calreticulin has been shown to be expressed on macrophages (51, 71, 72), as well as AML cells (12, 104). In phagocytic synapse (**Figure 2**, right side), the binding of signaling regulatory protein alpha (SIRPα) on macrophage to CD47 on AML (or other tumor cell) results in inhibition of phagocytosis (70). This elusive mechanism facilitates tumor escape by inhibiting phagocytic clearance of the tumor cells by the immune system. This process acts similarly to the PD1/PDL1 inhibitory pathway. Recently, it has been proposed that LRP1/Calreticulin/CD47 axis along with TSP1 are also implicated in T cell biology, specifically in T cell adhesion and motility in antigen-induced T cell stimulation (95, 96). Our findings show that interaction of LRP1 on T cells with membrane-bound Proteinase 3 (mP3) on neutrophils or AML in *ex vivo* settings leads to inhibition of T cell proliferation, suggesting that LRP1 is directly involved in T cell proliferation (12). Our preliminary findings in MHC-mismatched induced GVHD mouse models show that LRP1- deficient T cells fail to trigger GVHD in recipient mice, also suggesting an important role of LRP1 in T cell function (13). Overall, we believe that there is a lack of knowledge on LRP1 biology in various immune cell compartments. By drawing parallels with known molecular mechanisms, such as in phagocytosis between macrophages and tumor cells, we might be able to map out a new LRP1 mechanism to reveal its function(s) in T cells.

## Author contributions

JM, OS, LS, and QM substantial contributions to the conception or design of the work; or the acquisition, analysis, or interpretation of data for the work; drafting the work or revising it critically for important intellectual content; provide approval for publication of the content, agree to be accountable for all aspects of the work in ensuring that questions related to the accuracy or integrity of any part of the work are appropriately investigated and resolved. All authors contributed to the article and approved the submitted version.
